# Advancing digital health in low-middle-income countries: experiences from Armenia's health system digital transformations

**DOI:** 10.3389/fpubh.2026.1827775

**Published:** 2026-09-04

**Authors:** Tsaghkanush Sargsyan, Anna Artsruni, Sergey Sargsyan, Ruzanna Movsesyan, Arpine Muradyan, Avet Manukyan, Karine Sargsyan

**Affiliations:** 1 Digital Health Department, National Institute of Health Named After Academician S. Avdalbekyan, Ministry of Health of the Republic of Armenia, Yerevan, Armenia; 2 ARMED LLC, Yerevan, Armenia; 3 Arabkir Medical Centre – Institute of Child and Adolescent Health, Yerevan State Medical University, Yerevan, Armenia; 4 Department of Physical Rehabilitation, Armenian State Institute of Physical Culture and Sport, Yerevan, Armenia; 5 Office of Vice Rector for Research, Medical University of Graz, Graz, Austria; 6 IBD Institute and OncoBiobank, Cedars-Sinai Medical Center, Los Angeles, CA, United States; 7 Department of Digital Medicine and AI, and Department of Medical Genetics, Yerevan State Medical University, Yerevan, Armenia

**Keywords:** health digitalization, health technology disparities, healthcare digital literacy, low- and middle-income countries, mHealth interventions

## Abstract

**Background:**

Digital health systems and the implementation of those systems in low- and middle-income countries (LMICs) can measurably enhance and facilitate systematic healthcare efficiency; though, it is often challenged by infrastructural and socioeconomic limitations. Republic of Armenia has recently fast-tracked healthcare digitalization as part of broader health system reforms.

**Objective:**

To describe experience and lessons learned from the implementation of the country-level E-health system, including barriers and solutions relevant to LMIC settings.

**Methods:**

A review of National policies, established digital health infrastructure, and existing practices; data were also gathered from country health system reports.

**Results:**

The full-scale roll-out of the national-level electronic health network, called ARMED, empowered the extension of digital healthcare services—including all reporting systems, e-prescription workflow, registries, screening programs, and telemedicine. Implementation improved continuity of care and access to services, particularly in rural areas. Major challenges included fragmented health information, limited infrastructure, workforce and digital literacy gaps, and persistent rural–urban inequalities. Addressing these barriers required centralized governance, regulatory reforms, workforce training, and context-specific implementation approaches.

**Conclusion:**

The experience of Armenia can serve as an evocative guide, with practical steps for LMIC countries to fill gaps and tackle challenges. Experience in digital health governance can help fill gaps and strengthen the ability to use one's own systems to achieve health equity. The experience of Armenia clearly shows the impact of sustained governance, infrastructure development, and inclusive implementation strategies as critical factors for functional digital health implementation and transformation from paper to e-health in LMICs and can act for other countries pursuing equitable and scalable digital health integration as a well-documented case study.

## 1 Introduction

Digitization of healthcare is a major global priority and an essential component of health system modernization reform (1). Digital health technologies, including mobile applications, Electronic Health Records, and telehealth, possess substantial potential for improvement of healthcare access, continuity of care, health system efficiency, and population health outcomes (2). There are major possibilities to improve health outcomes by implementing programs that leverage medical technologies. Digital health provides citizens with access to better-quality services and enables physicians to deliver high-quality care (3). For example, mobile Health has the potential to improve medication adherence (4), and telemedicine improves access to and outcomes of care (5). These practices have been widely explored during the recent COVID pandemic (6), which accelerated the adoption of telehealth and digital care models worldwide.

The implementation of digital health systems in Armenia occurred in a non-linear process, even though the steps appear linear above for understanding purposes. Several steps occurred simultaneously, and there were several delays caused by administrative, leadership, and funding changes, legislation, and even shocks such as the COVID-19 pandemic and regional conflicts. Sometimes, it was necessary to go back to an earlier step for some projects. It is normal in developing countries. Below are the steps that occurred (1). In LMICs, limitations in digital and non-digital infrastructure, as well as low digital literacy, can exacerbate existing inequalities and be major obstacles in the process of adoption of inclusive digital health technologies. Exploring options and models, and analyzing practices and their outcomes, is an ongoing process that can be beneficial for expanding digital health, especially in resource-limited settings (3).

Armenia represents an important example of digital health transformation in a resource-constrained setting. Following major socioeconomic transitions after independence in 1991, Armenia implemented broad healthcare reforms and was classified by the World Bank as an upper-middle-income country in 2018 (7). The country has a strong historical background in information technology and a highly literate population, which created favorable conditions for digital innovation in healthcare.

Armenia's shift serves as a great model for others looking to integrate digital health fairly and effectively. They tackled the fragmented healthcare issue by launching a national electronic health platform, ARMED, that covers a wide range of e-health programs. The goal was to boost access to healthcare, ensure smoother, continuous care, improve data management, and provide more equitable healthcare, especially in rural and underserved areas. This paper lays out Armenia's journey in rolling out these digital health systems—how they developed the ARMED platform, incorporated telemedicine, and shared key hurdles they faced and lessons learned. Other lower-middle-income countries hoping to digitize their health sectors could find these insights super useful.

## 2 Methods

The research adopted a qualitative descriptive research design involving document analysis, national policy review, and key informant interviews. Document analysis included the systematic review of national policies, governmental decrees (e.g., Governmental Decree #95N dated 26 January 2017), reports by the Ministry of Health, documentation by the ARMED platform, and international sources (World Bank, WHO) for the period 2015–2026.

Semi-structured interviews were conducted with key informants involved in the digitalization process, according to the interview questionnaire in Appendix 1. Key informants included representatives of the Ministry of Health, the National Institute of Health, the ARMED operator (NEO), and implementing partners. Interviews covered the implemented processes, information systems, barriers, necessary support, and regulation aspects. With participants' consent, interviews were audio-recorded, transcribed, and content analyzed.

No identifiable individual patient data were used in this study.

## 3 Socioeconomic and demographic context

The Republic of Armenia is a small, landlocked country with a territory of 29,743 km^2^ and a population of nearly 3 million. Located south of the Caucasus Mountains, it borders Turkey, Georgia, Azerbaijan, and Iran. Armenia has a long and complex history; the territory of modern republic is only a small portion of historical Armenia. In 1828, the northeastern part of the Armenian Plateau was incorporated into the Russian Empire, and in 1920 Armenia became one of the republics of the Soviet Union. During the First World War, more than 1 million Armenians were killed in the Ottoman Empire, while many survivors emigrated and established Armenian diaspora communities worldwide. ^1^

During the soviet period, Armenia had developed an advanced industrial and scientific base, supported by strong research and engineering capacities. Armenia was one of the Soviet Union's leading centers of information technology. The first general-purpose computers were developed there in the early 1960s, and, according to estimates, one-third of the Soviet military's computers were designed in there.^2^^,^^3^

After declaring independence in 1991, Armenia became a member of the post-Soviet Commonwealth of Independent States, simultaneously pursuing closer cooperation with European community, becoming a member of the Council of Europe and the European Union's Eastern Partnership, like other post-Soviet countries. Armenia is part of the World Health Organization (WHO) European Region. ^4^

In the 90s, the country experienced a severe socioeconomic crisis caused by disruption of economic ties, the change of strategic tactics from a centrally planned to a market economy, armed conflict with neighboring Azerbaijan, and regional transport blockades. These causes major shortage of electricity, fuel, and food industrial decline consecutive unemployment, worsening living conditions, and significant emigration. Beginning in the early 2000s the country's economy has been on a continuous growth track since the early 2000s, interrupted by the world crisis in 2008, the COVID pandemic, and renewed armed conflict in 2020. Despite all difficulties and largely due to its inherited human capital and technological capacity, as a result of the positive economic trends, between 2000 and 2005, Armenia progressed from “low” to “lower middle-income country” and, due to further growth, was classified by the World Bank (WB) as “upper middle-income country” in 2018 (7).

According to WB estimates, in 2024, the Gross Domestic Product (GDP) per capita reached 8,556 current US dollars and 22,823 in purchasing power parity PPP International US dollars with per-capita GDP growth estimated at 3.5%. However the socioeconomic challenges persist, it's important to mention that the poverty rate was 23%, with 2% in extreme poverty. The Gini Index was 27.2, and the unemployment stood at 8.3%.

Another challenge ideography: emigration and low fertility rates bring to reduction of population. from 3.7 million in 1991 to 3 million in 2025. One-third of that decreased population were inhabitants of rural areas, which is a disproportionate share. Annual births decreased from nearly 80,000 to 34,000 in 2024 and the total fertility rate from 2.6 in 1990 to 1.7 in 2024. Thus, Armenia currently classifies in the group of countries with fertility below the replacement fertility level. At the same time, educational attendance of the Armenian population is high, and adult literacy rate is close to 100%.

## 4 Health care in the country

Armenia's health system was historically based on a model that followed central planning and was exclusively state-owned and state-financed. Following independence, the health sector underwent substantial reforms alongside the country's broader economic transition. Today, the Armenian health system combines elements of the former Semashko model with private-sector provision and emerging insurance-based financing mechanisms.

The system includes state-owned, region- and community-owned, private, and corporate healthcare facilities. The overall governance remains partially centralized but it facilitates the implementation of various health-sector reforms and initiatives. The Ministry of Health (MoH) is responsible for overall policies, regulations, and planning. There are few agencies under MoH such as the State Health Agency (SHA) responsible for technical management of the state health finances, the Center for Disease Control (CDC), caring for diseases prevention, the National Institute of Health NIH which works on statistical reports, regulatory documents, continuous staff education training and their licensing, as well as other institutions (health facility's licensing, quality control etc.). As an owner, MoH controls several large, specialized tertiary-level hospitals in Yerevan, the regional hospitals, and the National Ambulance service.^5^ Also, the state-owned key educational institution, Yerevan State Medical University, manages three hospitals and some other clinics. Since independence, many hospitals in Yerevan, the capital city, have been privatized, and many new private hospitals have been constructed. Most Primary Health Care (PHC) facilities, larger Policlinics in cities, and Family Doctors' offices in villages are still owned by the regions or communities. Still, some private institutions providing outpatient care have been established. Pharmacies and almost all dental services are now private, and there are many new private laboratories and diagnostic services.^6^ In total, there were 119 hospital facilities units with 11,963 beds in 2024. The number of beds per 1,000 of population is 4.3, which is lower than the WHO European Region indicator (5.2) but higher than in the group of WB's middle- and high-income countries (3.8). 438 units provide outpatient care (policlinics, family doctors' offices, etc.).^7^ There were 12,899 licensed and acting doctors (4.2 per 1,000) in 2024; this ratio is close to that of the EU (4.1) but higher than in the WHO-Euro and the upper-middle income countries' group (2.7 and 2.2). There are 16,844 paramedics and nurses (5.4 per 10,000). As above, by this indicator, Armenia is ranked between the EU (9.3) and upper-middle-income countries worldwide (3.8).

Despite the doctor-to-population ratio being adequate, a subnational proportion of health staff is concentrated in Yerevan, while regions and, mainly remote ones, experience a severe shortage of healthcare staff. Also, recruitment of young doctors into PHC facilities, family medicine, ambulance care, and rural practices is limited, resulting in workforce aging. Particularly, proportion of family physicians aged 65+ increased from 10 to 15% between 2008 and 2012. Thus, many PHC staff are overburdened by their workload. To tackle this problem, some initiatives to support positive development in the PHC system in rural areas have been launched (8).

## 5 Health financing

Following independence, the public spending on healthcare dramatically declined due to the economic collapse. According to World Bank estimates, after the 10 years of independence in 2000, overall per capita health expenditures were as low as 25 US dollars of which 4 US dollars represented public expenditure. At this time, support from United Nation agencies, international organizations, bilateral donors, and the Armenian diaspora played a substantial role in sustaining routine workflow and initiating health care reforms, including digitalization initiatives.

Nevertheless, over the past two decades, health expenditure in Armenia has increased substantially. Current health expenditure represent 9.96% of GDP; the per capita indicator is 766 current US dollars. Core PHC services and emergency services have been free for all population groups since the early 2000s. Inpatient services for children under 18 are publicly funded, and so are services for several vulnerable population groups.

However, out-of-pocket health expenditures were very high, accounting for approximately 80% of all expenditures, one of the highest proportions in European countries (see text footnote 7). To increase access to health services toward universal health coverage, the Government, starting from January 1, 2026, started the introduction of universal insurance for larger groups of population, such as persons of age 65+, those with monthly salaries exceeding 200,000 Armenian drams (approximately US$536 as of January 2026) 2026.^8^

## 6 Brief indication to health indicators

Armenia, as a country in transition, faces a combination of challenges typical for both developed and developing countries. Although child mortality has declined substantially over recent decades, still remains higher than in most European countries. According to UN agencies estimates in 2023 the infant mortality rate (IMR) was as 9 per 1,000, exceeding the average the WHO European region (see text footnote 1) (9) but remaining below the average rate of the middle-upper income countries (see text footnote 7). The life expectancy at birth is 77 (78 and 76 in the abovementioned groups, respectively). The crude death rate is 8 per 1,000 (8 and 7, respectively). The leading causes of mortality are known and expectable, as in other similar countries: cardiovascular disease, neoplasms, respiratory and gastrointestinal diseases, and traumas.

## 7 Implementation of digital health in Armenia: non-linear process: step-by-step road to digitalization of health services in Armenia

The implementation of digital health systems in Armenia occurred in a non-linear process even though the steps appear linearly above for understanding purposes. Several steps occurred simultaneously, and there were several delays caused by administrative, leadership, and funding changes, legislation, and even shocks such as the COVID-19 pandemic and regional conflicts. Sometimes, it was necessary to go back to an earlier step for some projects. It is normal in developing countries. Below are the steps that occurred. The process was carried out in the following steps:

• Step 1. Early initiatives

In early 2000s, when the country's health budget was still dramatically low, with initiative of the MoH, World Bank (WB), and later with the support of the US Agency for International Development (USAID), the first system for healthcare data collection was developed and implemented. Some state-owned health facilities were equipped with computers, and training sessions were conducted to enhance staff digital literacy and operational capacity. In 2009, USAID funding enabled the development and deployment of a digital system to register visits, facilitate patient documentation, calculate indicators, and generate reports in PHC institutions.

• Step 2. Internal management systems in health facilities

Since the mid-2010s, the internal management systems were implemented in several large healthcare organizations in Armenia. Particularly among the first, the largest pediatric hospital, “Arabkir” Medical Center—Institute of Child and Adolescent Health, and one of the largest general hospitals, “Erebuni” Medical Center, pioneered in that and began developing their internal data management systems. Currently, most healthcare institutions either have their own internal management systems or use dedicated modules of the National e-health system (ARMED). Since developing the national health care electronic system (see below), integrating internal systems with the national system is critical. Integration is usually done by proprietary protocols, as none of the existing systems support international ones.

• Step 3. Establishing a national electronic healthcare system

As stated above, the process of digitalizing the health system at different levels began in the early to mid-2000s within the framework of various initiatives. Some models and programs were developed and implemented, but these initiatives were mostly fragmented. To tackle this challenge and achieve more comprehensive digitalization of the health system, the Government of Armenia and the MoH held discussions to establish the national electronic healthcare system (EHS). The concept was developed within the framework of the state loan from the International Bank for Reconstruction and Development (IBRD), targeted at modernizing the public sector. The Government launched a tender, which was won by “Sylex SARL” (Switzerland) and “Maysis Apahov” LLC (Armenia). The winners established the “National E-Health Operator” CJSC (NEO). Next, the Government and NEO signed the concession agreement, under which the Government handed over management of the electronic health system (ARMED) to NEO for 15 years, pursuant to Governmental Decree of 26 January 2017 #95N. ^9^

Due to the lack of legislative regulations, ARMED was initially used solely to record visits that included the provision and financing of services covered under the Basic Beneficial Package guaranteed by the State. In addition, the private insurance companies providing the benefits were also connected to the ARMED. With the implementation of the electronic system, it has also become possible to collect health data and finance the process more effectively. However, extending electronic records leads to the optimization of the legal framework. Therefore, to expand digitalization of the health system, the country's parliament adopted special amendments to key national health legislation, such as the Law “On Medical Care and Services for the Population,” in May 2020. Initially, the Law defined the EHS. According to this definition, the system collects all healthcare data gathered during provision of healthcare services to patients. This means the system can include all data related to a patient, such as medical history and treatment, electronic prescriptions, electronic referrals, electronic sick leave certificates, vaccinations, and more. ^10^

To support further implementation, the MoH has developed and approved a series of sub-legislative acts, such as the “Procedure for entering and updating data on patients' visits, provided services, medical interventions, diagnoses, prescriptions, and personal health data, including special category data, in the EHS,” the MoH decree 40-N (2021). This decree defines the “Rules for Viewing Patient Data Stored in the Electronic Information Window of the EHS,” the scope of authority for persons accessing information with the informed consent (IC) of the patient (or their legal representative), the procedure for accessing the patient's electronic healthcare record, viewing their lifelong medical data, and the consent form required for the patient and practitioner to access the electronic system. In accordance with other regulations, these acts govern data collection processes and regulate access to and permissions for the patient's profile.

• Step 4. Provision of equipment

In 2019, the Global Fund financially supported the MoH to procure and distribute 2420 laptops to medical organizations, providing at least one computer per PHC office and hospital department. Following that, NIH launched a series of training sessions on data entry skills for 1,800 PHC physicians; due to the COVID-19 pandemic, the training sessions continued online (less effective than in-person training). In 2024, the MoH, with financial support from the Global Fund, procured 120 all-in-one computers and distributed them to medical organizations in remote regions. In 2025, rural nurse points (small cabinets where the nurse is the only employee) were equipped with tablets and portable ECG devices to enable telemedicine with permanent connection to doctors.

• Step 5. Staff training

To strengthen the knowledge of the healthcare professionals, in 2025, financed by the known Armenian Diaspora's Hovnanian Foundation, 1,200 doctors were retrained on digital health and digital data, the accuracy in decision-making, digital architecture, health care architecture, comparison of standards, applications, data management, reporting, anomaly identification, and correction. and so on, in addition to practicing selected skills in the ARMED system.

To facilitate the further and sustainable functioning of NESH at the facility level and to help doctors and nurses, a decision was taken to establish, in health facilities, positions of focal “operators” who served as points of contact for digitalization. These operators had undergone high-level training in the following topics: digital health, ARMED, disease registries, ICD-10 and ICD-11, their advantages and disadvantages, management tools, artificial intelligence, telemedicine, legal regulations, data protection, interoperability, privacy, data exchange, and cybersecurity.

• Step 6. Defining interoperability and standards

At present, several country-level registries are being established and managed within the ARMED system, including the medicines registry, the healthcare professionals' registry, the medical organizations registry, and others. All licensed medical organizations are required to connect to ARMED and exchange data through internal interoperability mechanisms. Within ARMED, patient identification is ensured through integration with the State Population Register (SPR), and data is retrieved using the Public Services Number (PSN). Diagnoses are recorded according to ICD-10, while the international LOINC standard is used to standardize laboratory tests.

The system is integrated with the Government Interoperability Platform and receives data from other public authorities' databases, including personal data from the State Population Register, disability-related information, kinship data from the Civil Status Registry (CRVS), data from the Ministry of Education, and other relevant sources.

• Step 7. Developing and implementing NEHS components

All components required to form a citizen's electronic health record (EHR) were implemented within the ARMED system.

° Sub-step 7.1. E-referrals

The first successful and best practice was the implementation of electronic referrals. It was successfully introduced at the beginning of 2020 and has since continued to develop and improve, ensuring fast, high-quality delivery of medical services.

° Sub-step 7.2. E-prescription

The development and piloting of the electronic prescription system was done as early as 2018; however, it was fully implemented for all state-financed medicines in 2022. Prescription-only medicines need a mandatory digital prescription. Consequently, the e-prescription system was launched on March 1, 2024, for commercial pharmacies as well and is mandatory for all prescription-only medicines. This provided visibility and accountability of medication costs and enabled the issuance of electronic prescriptions to citizens. E-prescriptions are accessible in the ARMED mobile application or through an SMS code sent to the patient at the moment of issuing the prescription.

There is a challenge in having a more flexible mechanism for electronic prescriptions. The drug registry is maintained within ARMED, and updates to the lists are carried out manually: data is received via official correspondence and entered into the system, which delays practical use of newly registered medicines and sometimes causes technical problems. Since 2020, the Government, in cooperation with USAID, has developed the technical specifications and system design for the digitalization of the medicines registry and the importation and registration processes for medical devices.

However, due to the project's scale and emerging operational issues, the system has not yet been implemented, and the process continues as described above.

° Sub-step 7.3. Sick leave certificates

The full implementation of sick leave certificates took place on September 1, 2025. It involves data exchange between the information systems of three government bodies. The ARMED electronic health system, where the physician creates and manages the sick leave certificate for the patient; the Ministry of Labor and Social Affairs system, where benefits are calculated based on data received from ARMED; and the State Revenue Committee's information system for employers to access the employee's sick leave count and information.

° Sub-step 7.4. Vaccination records

The entire immunization process, including vaccination and post-vaccination adverse events, is fully digitized. The immunization schedule has been fully implemented, and a vaccination record is automatically generated for each resident. The digitization of historical data is ongoing, with issues arising from missing or incomplete paper records due to archiving problems at health facilities. Nonetheless, the available data was extensively digitized, allowing citizens to access their vaccination records through a mobile app. Currently, a digital certificate with a QR code is available on both the ARMED platform and the patient's mobile app. The National CDC manages the process.

° Sub-step 7.5. Virtual warehouses and logistics systems

The process of managing the storage, movement, receiving, issuance, and remaining stock of vaccines and medicines has also been implemented in ARMED. Each organization has access to its own virtual warehouse data and can monitor both incoming and outgoing stock. Outflows are mostly automated, with quantities adjusted based on information entered about individual vaccinations or medicine dispensing. The entire flow of vaccines, humanitarian and state-funded medicines is carried out from a virtual central warehouse to individual organizations' warehouses.

• Step 8. Implementation of registries

° Sub-step 8.1. Served population registry

This was the first registry created within ARMED. It ensures the processes of population, enrollment, arbitration, and reporting. At present, all primary healthcare facilities use this registry to provide services to the enrolled population. It is also the basis for financing.

° Sub-step 8.2. Clinical registries

Clinical registries are built seamlessly based on the clinical data collected for each patient. After stating any diagnosis included in the registry, the doctor needs to fill a small document with additional data. This includes general data along with data available in the patients' record forms, as well as the country-level registry. However, practice has shown that there are certain problems with the correct use of ICD codes. Currently, registries are developed for diabetes mellitus, malignancies, stroke, and mental health. The tool data analyses and report generation.

° Sub-Step 8.3. Blood donors' registry and blood registry

The digitalization of blood registries has started recently. It has primarily been conducted in collaboration with the Yeolyan Hematology and Oncology Center and other centers; donor banks have been integrated into the centralized bank within ARMED. The process of including all blood banks in the country is underway. Still, legal amendments, government decisions, and ministerial orders need to be revised or aligned to determine their appropriateness and to ensure efficient functioning at the country level.

° Sub-step 8.4. Organ registry

A registry of deceased donors (liver and kidney) and recipients has been established, with all required legal frameworks in place.

° Sub-step 8.5. Sperm registry

The sperm registry is basically managed in the same way as the donor registry. Artificial insemination is currently prevalent, and excessive insemination from the same sperm source could lead to issues related to marriage in the future. To overcome these issues, the system records the donor and imposes restrictions. For example, if children are born from that sperm in three to five families—taking into consideration that the important factor is the number of families, not the number of children- further use is limited. Additionally, a future concept could involve individuals seeking to marry by submitting a compatibility test. The system would flag an incompatibility without disclosing the specific reason, as this constitutes confidential information that must remain inaccessible to unauthorized individuals.

° Sub-step 8.6. Cornea registry

The creation of this registry became a priority following the 44-day war. The registry was established, but due to legal regulations and issues with corneal imports, its implementation was delayed.

• Step 9. Digitalization of screenings

The National Health strategies are targeted at the prevention and prophylaxis of various non-infectious and other diseases, particularly through regular health screenings. Thus, to improve management and accountability for screening results, these activities have also been incorporated into ARMED.

For example, since 2021, the breast cancer screening has been launched in regions using a mobile mammography unit, and it has been a successful program. During this program, the first large-scale collection of clinical data began. To improve the process quality, the concept of telemedicine was applied: the technician in the region performs the examination and uploads the image in DICOM format to the appropriate server. From there, doctors in Yerevan review the image and make the diagnosis, which then becomes available to the primary healthcare physician and the patient through the system. At present, static mammography units across the entire republic have also joined this program, and screening is being carried out on a larger scale, covering more women in the relevant age group.

Since 2025, screening for cardiovascular diseases and diabetes has also been conducted through ARMED. These three screenings are now carried out using a new methodology that actively involves patients. The patient completes an initial questionnaire in the app and is automatically registered for mammography or another screening service.

• Step 10. Introduction of telemedicine

The telemedicine component, as mentioned above, was first applied during mammography screening, when the patient is in a region while the physician is in Yerevan, and the medical service is delivered remotely. In 2024, the next telemedicine component was introduced: third-level maternity hospitals in Yerevan began providing telemedicine consultations to second-level maternity hospitals in the regions, with the entire process—including data collection—being carried out through ARMED. In 2025, rural nursing posts in remote regions were also included in this process, since a nurse is permanently present at these stations, whereas a doctor visits periodically or as needed. This creates challenges related to the efficient use of time and transport, for which telemedicine is the best solution.

So far, the tool has been piloted in 20 rural nursing posts, where the first 20 tablets were provided. As noted above, an additional 310 tablets have already been distributed to other sites, and distribution of ECG devices is also underway. The legal regulation of telemedicine was approved in 2022.

• Step 11. Statistics and reporting

At present, ARMED contains a considerable number of reports and registries, from which data can be obtained and analyses conducted. Certain dashboards make the data more visible and easier to interpret. However, for the NIH National Statistical Information Center to receive all necessary official statistical reports, significant corrections and improvements are still needed. This work is in progress. These reports are an important part of the toolkit that will help make the database more accurate and trustworthy.

• Step 12. Electronic health records

As already mentioned, there are internal management systems (EMR) and the national electronic health system (EHR). To complete the health history for each patient, relevant information from internal management systems must be transmitted to ARMED. According to national regulations, upon completion of any service, health facilities are obliged to provide a final clinical report. At present, the goal is to mandate the digital completion of this report and its transmission to the ARMED platform.

Currently, the workflow is realized and operational. A remaining issue with standardization and on spot—clear formulation of legal regulations, specifically defining a mandatory dataset that must be included is still ongoing.

• Step 13. Mobile health application

The ARMED mobile application was created and implemented during the COVID19 pandemic. In fact, at that time, ARMED launched the digitalization of the entire pandemic management process—from testing to triage —very quickly. The red and green QR code feature in the ARMED app, showing the test result status, made citizens' lives easier during this difficult period. The QR code also gained recognition by the UN and was used during travel. Armenia was the first country to have this version.

At that time, people were using the application extensively, but after the pandemic, interest declined. Nowadays, the application is once again attracting significant interest as a valuable tool for citizens to stay informed about their health history, visits, services received, and documents such as prescriptions, referrals, vaccination records, and other certificates.

It also provides information on the status of the universal health insurance introduced in 2026, as well as available services and medications. The application is the most important tool for patient empowerment, enabling more accurate and reliable data, since citizens are best positioned to monitor and control their own information.

A citizen can grant permissions to view their data through the application in accordance with “Personal Health Data, Including Special Category Data, in the EHS”, MoH Decree 40-N (2021), which defines the “Rules for Viewing Patient Data Stored in the Electronic Information Window of the EHS, the Scope of Authority of Persons Accessing Information with the IC of the Patient (or their legal representative), the Procedure for Accessing the Patient's Electronic Information Window and Viewing Their Personal Data, Including Special Category Data, as well as the Form of Consent for the Patient to Access the Electronic System”. As a result of these efforts, a patient can schedule a virtual appointment with their doctor and receive a telemedicine consultation.

A mobile application enables patients to complete the appropriate questionnaire and register for breast cancer, diabetes, and cardiovascular disease screenings.

• Step 14. Centralized emergency medical services dispatch system

In 2013–2014, a computerized information system for managing and recording emergency calls was introduced in Yerevan and ambulance stations. The system included Armenia's first interactive map, enabling ambulance vehicles to use geolocation and navigation. Improved in 2016 and further enhanced in 2025, the dispatch system is currently implemented across all ambulance services in Armenia, involving more than 123 stations and 250 computers. It relates to ARMED and the State Population Register. The system guarantees end-to-end observing of emergency calls with help of implemented automated call recordings and real-time services: ambulance vehicle tracking, case status monitoring, and operational dispatch management. Currently, it is serviced by a private company, commissioned through a tender competition.

• Step 15. Health workforce registry

In 2023, the Healthcare Workers Registry was created and implemented, which currently includes 80% of healthcare workers in Armenia. The registry contains information about a healthcare worker's education, professional competencies, and credits. The registry's database is used for the certification and licensing processes for healthcare workers. At present, it is not integrated with ARMED, but it is planned that this registry will serve as the primary data source for ARMED, allowing permissions to be assigned by specialty and errors to be avoided.

• Step 16. Licensing registry

Until 2023, licenses in Armenia were issued either indefinitely or for long-term periods. In 2023, it was decided to issue licenses for a 5-year term to better control and manage subsequent processes. A digital registry will provide more accurate data and enable more effective administration.

The development of Registry of Licensing started in 2024, comprises the provision of medical services and is now ready for official launch. Legal entities submit an application, and all necessary data is obtained through the interoperability platform. The system automatically verifies and approves the license, without human intervention. The registry also manages subsequent processes, including suspension, revocation, and reissuance of licenses.^11^

• Step 17. Birth and death registration

Birth and death registration in Armenia is carried out by the Civil Status Acts Registration Authority (CSARA), which acts under the auspices of the Ministry of Justice. As a part of public service improvement and to provide citizens with services under the “one-window” principle, the civil status registration process has been digitalized. In the ARMED eHealth system, medical certificates for births and deaths are currently registered by the relevant healthcare professionals. Afterward, through ARMED, the data are transmitted to the CSARA databases, and the system receives the official birth or death record, which is then issued to the citizen directly at the healthcare facility.^12^

• Step 18. Laboratory registry

The MoH, together with ARMED, has translated and localized the LOINC standard, and it has already been mapped to all laboratory standards and methods used in Armenia.

Many medical institutions already have laboratory and diagnostic (instrumental) examination (LIS) systems integrated into their internal management systems. These internal management systems are linked with ARMED; however, the availability and access to this data within ARMED have not yet been fully ensured. ARMED itself is also connected to some of these LIS, but the process remains incomplete and not fully regulated. Some medical institutions lack the financial capacity to address these issues. Regional medical centers still lack their own internal management systems or LIS. Polyclinics also face difficulties, as they mostly depend entirely on ARMED.^13^

The MoH has taken certain steps to advance this process. An agreement has been reached with one of the service providers to implement LIS in regional medical centers at relatively affordable prices. For polyclinic organizations, this will be addressed by centralizing services—meaning polyclinics will outsource them to a centralized laboratory, which will provide the information in digital form. Nevertheless, ARMED includes laboratory test referrals and allows manual data entry.

• Step 19. Drug and medical equipment registry

The “Expert Center for Drugs and Medical Technologies” State Non-Commercial Organization (SNCO) currently operates its own system that serves as the registry of registered medicines.

During the implementation of the electronic prescription system, it became clear that the system could not be used as a digital data source due to data issues. As mentioned above, ARMED therefore created and began using its module solution.

In response to the demands of the time and to digitize the entire process—from the import of medicines and medical devices to their registration—the Government of Armenia launched a digitization initiative for this function. Although the process has not yet been completed, certain functionalities—especially those related to medical devices—have already been initiated (see text footnote 13).

• Step 20. International integrations in eHealth and telemedicine

Alongside the government's interoperability platform, discussions are ongoing within the frameworks of the European Union's EU4eHealth, EU4Digital, and EU4EaP, as well as the WHO and the Eurasian Bank's programs for member countries, regarding the integration of electronic health systems and collaboration in telemedicine (10).

• Step 21. Life events program

The Government of Armenia is implementing a Life Events Program to digitalize life events (see text footnote 5). As part of this initiative, the Ministry of Health is involved in two events: “Childbirth” and “Maintain Health.” The “Childbirth” three-in-one program covers the entire process from a child's birth through official registration, pediatrician enrollment, and related benefit arrangements. Under the “Maintain Health” event, plans include supporting access to medical services throughout a person's life—from birth to death—while encouraging citizen participation through digital platforms (e.g., submitting applications and providing feedback or satisfaction surveys), in line with the one-window principle. Currently, the “Childbirth” event is in the final stages of implementation, while “Maintain Health” is actively underway (11, 12).

## 8 Discussion: lessons learned

In the digital health transformation in Armenia, there are so many facets that this qualitative study can only briefly illuminate, especially given that the process is very dynamic and changes occur almost every month. Given the broad scope of the presented interventions and the lack of scientific evidence for each, the authors don't claim to provide a comprehensive analysis. Nevertheless, enough lessons have been learned to be useful for implementing such programs in Armenia and in countries with settings that are more or less similar (13). Like any other innovative idea, digitalization encounters its own challenges, and that was the case in Armenia. In terms of conceptual understanding, digitalization has been widely used to increase the efficiency of health service provision. Nowadays, any initiative aimed at improving the quality of health care, such as national or local programs to optimize the work of PHC-level facilities or hospital services, should include a digital component. Digitalization will inevitably continue to penetrate various aspects of health care and affect the work and lives of every care provider and caretaker from birth to death. Continuous development and integration of electronic tools leads to the idea of having a “digital health passport” for every person, which may play a crucial role throughout the lifespan (14, 15).

To be more effective and comprehensive, digital health information should be closely integrated with the digital systems of other sectors, such as social or justice sectors. Our experiences revealed some challenges with these integrations, as each sector at some stage started developing its own “separate” system. The latest government-led project, like the Life Events Program, exemplifies this effort.

The ethical issues remain very significant, including proprietary systems that jeopardize data privacy and varying levels of access that lead to patients' loss of control over their data, highlighting the need for strong governance. The Armenian Government placed special emphasis on consent-based access, along with regulatory changes such as the 2020 updates to the Law on Medical Assistance and Services, which align with the WHO Global Strategy on Digital Health, which advocates for ethical and secure data stewardship (see text footnote 4) (15).

Armenian experiences and the complexity of tasks related to comprehensive digitalization of healthcare clearly indicate the need for a broad, participatory approach to developing effective digital tools with the involvement of all key stakeholders: health managers, including those engaged in health policies, planning, and financing, as well as doctors, nurses, IT professionals, and statisticians. Such an inclusive approach was not always practiced in Armenia. Conversely, a fragmented approach can lead to reduced effectiveness and wasted resources. Therefore, careful, detailed planning of implementation steps and areas in digital health, as well as stakeholder involvement, should be a top priority from the beginning (16, 17).

The implementation of the electronic health system necessitated significant revisions or amendments to basic laws and government decrees governing the provision of health care. In some cases, the lack of such regulations slowed practical action. Therefore, a very deep and comprehensive analysis of the regulatory framework is needed prior to the initiation of digitalization programs.

Implementing such a large-scale project requires significant resources. In the Armenian case, support from international actors was crucial. The same may apply to any other country with limited resources. Therefore, international health programs (such as WB or Asian Bank programs) as well as various bilateral projects are important, especially for initiating the process. Also, as noted above, any contemporary health project should include relevant digital components.

As was expected, the current status of the health system, its weaknesses in the pre-intervention phase, complicate the process of digitalization. This includes gaps in legislation and other regulations, a lack of resources to provide necessary equipment, inadequate staffing, and the perceptions, attitudes, and capacities of health workers to accept and implement innovations. These challenges should be taken into consideration while planning the process. Nevertheless, the rational digitalization should be targeted and can mitigate existing challenges.

Reforming the health care system is a continuous process typical not only of Armenia. On one hand, this is a problem, as working in a static environment could be easier. But from another perspective, this is an opportunity if digitalization processes are aligned and organically incorporated into reforms, serving as facilitators of those reforms.

Cultural and mental aspects are important: doctors, especially those in old age, are accustomed to working with paper. As Armenian experiences have shown, in some places people rely on paper versions of the necessary data rather than accessing them on computers. This is a matter of culture and knowledge, which is quite challenging to change. However, doctors, willingly or not, have started to learn and adapt, while management and administration still face this issue.

## 9 International experiences

Implementing fully developed national digital health platforms is highly important, and possibly essential, for health systems transformation and for maximizing digital health investments in low- and middle-income countries (LMICs). Recognizing the importance and timing of these platforms supports ongoing monitoring and evaluation. Clearly defining the components, functions, and governance of these platforms is crucial for identifying priority use cases related to expected health impacts in selected countries (18). Proposed performance indicators allow for initial impact assessments before and after platform adoption. Although LMICs share similar development needs, government health priorities can vary significantly, and the political economies involved in platform implementation differ widely. Analyzing regional health outcomes, governance measures, and digital health contexts helps identify key factors in selecting health system components (19).

The health objectives, health challenges, and political economy influences play an important role in the development and adoption of national digital health systems in LMICs. The priority for Sub-Saharan Africa is to combat the HIV/AIDS epidemic. Still, other health challenges are increasingly mentioned, such as maternal/child health, communicable disease outbreaks, non-communicable diseases, and universal health care coverage (19). Several constraints influence the use of digital health platforms in the region, such as financing for digital projects and the absence of vendor competition.

The systems of procurement, financing, knowledge sharing, and regulation show significant differences between LMICs closer to the World Bank's low-income threshold and those farther from it. Yet, the systems for sharing international data, mutual recognition of standards, and privacy protection remain scarce in the latter group of LMICs. Open-source health information exchange architectures are scarce in LMICs bordering middle-income countries; as a result, cross-border collaboration is low (20, 21). Following the wide adoption of health management information systems, sub-Saharan Africa has become the first region to scale open-source community health information systems. Innovative financing mechanisms to promote system user ownership and co-investment remain untested. The Health Pooled Fund and the Caribbean Community Regional Framework for Emerging Diseases and Health Security demonstrate regulatory as well as financial partnership, correspondingly, while the Southern African Development Community Health Community offers pilots that share the benefits of scale after prior implementation. By supporting regional partnerships, creating dedicated policy pages, and providing clear steps toward harmonization, national-level initiatives can help avoid costly duplication and adapt more quickly to emerging global trends (22, 23). Digital health investments in low- and middle-income countries (LMICs) have enabled numerous countries to adopt digital health platforms as a priority (19). Engaging national leaders and health stakeholders through a preliminary survey of the health information systems (HIS) ecosystem and a digital health platform can identify priority use cases for such platforms and map existing national strategies against the HIS assessment and country priorities. These platforms provide a set of technologies for collecting, managing, and sharing data; for example, nearly all priority platforms are linked to a comprehensive electronic health records strategy that aims to deliver integrated, person-centered health services along the continuum of care. An HIS ecosystem assessment can provide insights into enablers and barriers that support or constrain the effective functioning of these platforms (24). Key enabler areas include governance and stewardship; policy, legal, and regulatory frameworks; standards; human resources; infrastructure; data stewardship; and financing.

Enhancing health information systems is a priority under the Sustainable Development Goals, which has led to a surge of global interest in digital health. However, some LMICs have adopted digital health platforms and health information systems to improve service delivery, the quality of information provided, and the accountability of all stakeholders. The above are experiences from LMICs in developing digital health information systems, which can inform them in they develop their own systems. The national digital health platforms play a significant role in providing guidance on developing interoperable data standards, governance, and phased investment, aligned with national priorities and constraints (25). The experiences of the various LMICs have shown significant milestones in developing the national digital health platforms, which provide insight into the various approaches that can be used in governance, funding, as well as innovative ways of engaging stakeholders in delivering health care services. In each of these cases, a different approach has been used based on the specific demands involved in providing health services in that particular country. The examples discussed in this context would provide a wealth of information to other countries that wish to develop a health information system for themselves using digital technology (26). As presented in Appendix 2, there is a table that contains the country profile along with economic, health, and digital health issues. They illustrate how national digital health platforms are governed, funded, and scaled, with emphasis on interoperability, data governance, and stakeholder engagement across different sectors. Digital health is expected to play a major role in national health systems, and governments plan to introduce digital health and information technologies to augment existing systems (27). In lower-income countries, future digital health technologies are expected to revolve around mobile health applications, artificial intelligence (AI), social media to enhance community engagement, the incorporation of virtual reality sessions at primary health centers, digital twins for public health, blockchain for efficient service delivery, and connected devices to access vital information and deliver effective reminders (19). Emerging technologies and artificial intelligence are expected to transform health care worldwide. On the other hand, ethical data access, broader governance to protect data privacy, rights, and ownership, and active stakeholder engagement, more precisely with health professionals and patients, underlie the deployment of technologies. With the rapid deployment of technologies and the challenges of keeping pace with these advancements, questions arise about their sustainability and the mechanisms for mitigating risks (28). The literature shows a significant concern about the impacts and contributions of digital health and digital health technology on health outcomes and quality of service in lower-income countries, and research priorities and recommended indicators to monitor such contributions are highlighted. A technology-enabled health platform framework is proposed, in which contributions from a technology-enabled health system to effective health coverage, efficient health service delivery, and health systems strengthening are sought.

## 10 Conclusion

Only wide-ranging future research should and can assess the long-term effect of the process described above. The experience of Armenia demonstrates significant improvements in digital health equity, contributing to the achievement of Sustainable Development Goal 3 globally.

Armenia's experience in digital health transformation is highly relevant to LMICs striving to achieve health equity through the digitalization of the healthcare system. By addressing the issues of fragmentation, infrastructure, and digital literacy through the implementation of a set of coordinated reforms in the field of regulation and strategic investments in the field of health information technology, Armenia proves the potential of the integrated system in facilitating the streamlining of health processes, including e-prescription and telemedicine, in the best practices of other nations.

## Data Availability

Publicly available datasets were analyzed in this study. This data can be found here: www.armstat.am, www.moh.am, www.nih.am, hrh.am, armed.am.

## Footnotes

1 https://www.britannica.com/place/Armenia (Accessed March 5, 2026).

2 https://www.forbes.com/sites/craigsmith/2023/12/08/soviet-mainframes-to-silicon-mountains-armenia-as-a-tech-powerhouse/ (Accessed March 5, 2026)

3 https://www.armenianinstitute.org.uk/viewstext/computing-in-armenia (Accessed March 5, 2026).

4 https://www.who.int/armenia (Accessed March 5, 2026).

5 https://moh.am (Accessed March 5, 2026).

6 https://eurohealthobservatory.who.int/publications/i/health-systems-in-action-armenia-2024 (Accessed March 5, 2026).

7 https://data.worldbank.org/indicator/SH.MED.BEDS.ZS/ (Accessed March 5, 2026).

8 https://www.primeminister.am/en/press-release/item/2025/12/25/Cabinet-meeting//b (Accessed March 5 2026).

9 https://www.irtek.am/views/act.aspx?aid=88459 (Accessed March 5, 2026).

10 https://p4h.world/en/documents/law-of-the-republic-of-armenia-on-health-care-and-service-to-people-amended-in-may-2020/ (Accessed March 5, 2026).

11 https://health.e-gov.am/hy/service/medical_care_and_maintenance_ license/info (Accessed March 5, 2026).

12 https://hrh.am/am/auth/loginnew (Accessed March 5, 2026).

13 https://hartak.am/(Accessed March 5, 2026).
