# Supplementary material for: Advancing digital health in low-middle-income countries: experiences from Armenia's health system digital transformations
Source: Front Public Health. 2026 Sep 4;14:1827775. doi: 10.3389/fpubh.2026.1827775 (PMC13585739; doi:10.3389/fpubh.2026.1827775)
Supplement: Supplementary file 1 [file Supplementary_file_1.docx]

# Appendix 1

QUESTIONNAIRE

Qualitative research conducted within the framework of a master’s thesis on the topic of digital healthcare strategy in Armenia

a) Name/Surname of the Interview Participant
b) Position/Institution of the Participant
c) Interview Date: Day/Month/Year
d) Anonymous/ Non-anonymous
e) Recorded/ Not Recorded
f) Interview Duration (minutes)
g) PPlease describe the processes implemented within your organization or scope of activities, the extent to which they are digitized, the information systems used, the applied lists, their sources, and the regulatory normative acts.
h) Do you need to obtain information from other governmental bodies to perform your functions, and if so, in what format do you receive this information?
i) What obstacles do you face in collecting reliable digitized information? • Human resource skills • Technical resources • Communication capabilities • Inadequate software solutions • Regulatory frameworks • Other (please elaborate):
j) IIn the context of implementing comprehensive digital healthcare, what support does your organization need? • Business process analysis • Training • Technical upgrades • Centralized software solutions • Tools for information collection • Other (please elaborate):

Appendix 2.

**Countries and development state of digital health system**

**Country**

**Income Level (World Bank approx)**

**Key National Digital Health Initiative/Platform**

**Governance Model**

**Interoperability Approach**

**Scale-Up Strategy**

**Funding Mechanisms & Key Partners**

**Status/Notes (as of ~2020 onward, up to 2025-2026)**

**Source**

Albania

Upper-Middle

National eHealth strategy & platforms

Government-led, EU alignment

Interoperability standards

EU-supported rollout

Government + EU, donors

Transitional; Eastern Europe peer alignment

https://extranet.who.int/countryplanningcycles/planning-cycle-files/national-health-strategy-albania-2021-2030

Armenia

Lower-Middle

ARMED (national EHR and health information system)

Government-led (Ministry of Health)

Data synchronization, standards for clinical/admin/financial data

Phased rollout (2017 onward), full deployment targeted 2025

Government + international (e.g., WHO, NIH training, donors)

Advanced foundational; COVID/vaccine modules, patient access expansions; ISO certified

https://armed.am

Bangladesh

Lower-Middle

99DOTS & national health info systems

Government + innovative pilots

Mobile-based adherence & data tools

Targeted disease-specific to national

Low-cost innovative funding

Effective for TB adherence; broader mHealth push

https://www.who.int/publications/i/item/WHO-2018-99dots-tuberculosis-adherence

Bosnia and Herzegovina

Upper-Middle

Entity-level digital health initiatives toward national

Decentralized, multi-entity

Emerging national standards

Phased integration

Government + EU, international

Fragmented but advancing; post-conflict context

https://health.ec.europa.eu/system/files/2019-02/2019_chp_bosnia_herzegovina_en_0.pdf

Ethiopia

Low

National digital health strategy & platforms (e.g., DHIS2-based)

Government leadership & co-creation

Interoperable systems with donor alignment

Accelerated leapfrogging via partnerships

Government + international donors

Rapid progress; foundational infrastructure

https://www.moh.gov.et/site/sites/default/files/2021-01/Digital%20Health%20Strategy%202020-2025.pdf

Georgia

Upper-Middle

National eHealth strategy & platforms (e.g., integrated HIS, telemedicine)

Government-led with multi-stakeholder

Emerging standards for data exchange

Pilot to national, pandemic-accelerated

Government + UNDP, Sweden, international partners

Strong progress; e-learning for health workers, emergency/public health focus

https://moh.gov.ge/en/

Ghana

Lower-Middle

National digital health & vaccine delivery platforms

Government + international collaboration

Interoperable vaccine & health registries

Pandemic-focused digital innovation

GIZ, UNICEF, partners

Interoperable solutions advancing UHC

https://www.moh.gov.gh/wp-content/uploads/2021/07/Ghana-Digital-Health-Strategy-2020-2024.pdf

India

Lower-Middle

Ayushman Bharat Digital Mission (ABDM)

Strong government-led with private sector

National standards for health IDs, data exchange

Phased national rollout, integration with existing systems

Government investment + private tech ecosystem

Advanced; comprehensive EHR/telemedicine integration

https://abdm.gov.in

Kazakhstan

Upper-Middle

National eHealth strategy & integrated platforms

Strong government leadership

Interoperable systems, data sharing

Phased national investment

Government + international

Mature regionally; NCD management, digital skills emphasis

https://www.gov.kz/memleket/entities/dsm

Kenya

Lower-Middle

National digital health strategy (e.g., Kenya Health Information System)

Multi-stakeholder governance

Standards-based interoperability

Pilot to national scale

Government + PPPs

Notable for mHealth & community health tools

https://www.health.go.ke/wp-content/uploads/2016/07/Kenya-eHealth-Policy-2016-2030.pdf

Kyrgyzstan

Lower-Middle

National eHealth strategy & platforms (e.g., electronic records, telemedicine)

Government-led

Focus on mobile/digital tools for access

Youth-led innovations + national rollout

Government + UNDP, international

Growing; chatbots/apps for education, rural focus; PrEP guidelines updated

https://med.kg

Malawi

Low

Digital health initiatives (e.g., drone/data academy, eHealth)

Government with innovative partnerships

Data & logistics integration

Focus on access in remote areas

Donors & academies

Innovative for supply chain & data

https://www.health.gov.mw/index.php/resources/digital-health-strategy

Malaysia

Upper-Middle

National digital health interventions (e.g., MySejahtera, teleconsultation)

Cross-sector capacity-building

Integrated dashboards & apps

COVID-19 catalyzed rapid scale-up

Government + cross-sector stakeholders

Scaled during pandemic; sustainability lessons

https://www.moh.gov.my

Moldova

Lower-Middle

National digital health ecosystem (e.g., MCloud integration

Government-led, cross-sector

Interoperability via national platforms

Phased digital governance alignment

Government + EU/international donors

Notable for digital inclusion; integrated services, policy for literacy

https://egov.md

, eHealth services)

Mongolia

Lower-Middle

National digital health strategy & platforms

Government-led

Standards for health info systems

Phased, donor-aligned

Government + WHO, international

Comparable maturity; remote/rural delivery focus

https://moh.gov.mn

Nigeria

Lower-Middle

National digital health strategy & platforms

Federal + state governance

Emerging interoperability standards

Large-scale population focus

Government + donors

Growing ecosystem; focus on young populations

https://health.gov.ng/doc/National-Digital-Health-Strategic-Framework-2020-2025.pdf

Philippines

Lower-Middle

National digital health (e.g., 99DOTS expansion)

Government-led with cost-efficient tools

Mobile/SMS integration

Cost-effective scaling

Government + international support

Efficient for adherence monitoring

https://ehealth.doh.gov.ph

Rwanda

Low

National digital health ecosystem (e.g., OpenMRS, rapidSMS adaptations)

Government-led with strong political will & partnerships

Focus on interoperability & data exchange

Partnerships for leapfrogging tech adoption

Donor co-funding (e.g., USAID, Gates Foundation)

Success story; primary care transformation

https://www.moh.gov.rw/fileadmin/user_upload/MoH/Publications/Policies/National_Digital_Health_Strategic_Plan_2018-2023.pdf

Tajikistan

Low

Emerging national digital health initiatives (e.g., strategy alignment)

Government-led with regional support

Basic interoperability pilots

Donor-supported scale-up

Government + UNDP, WHO

Foundational; remote access emphasis, regional health security

https://moh.tj

Tanzania

Lower-Middle

National eHealth strategy & DHIS2 platforms

Government-led with regional focus

Interoperable district health systems

Phased, donor-supported

Donors (e.g., UNICEF, WHO)

Strong HIS foundations; sustainability challenges

https://www.moh.go.tz/storage/app/uploads/public/5d3/ea0/61f/5d3ea061f2c9b308344068.pdf

Uganda

Low

Telemedicine platforms & national strategies

Government + rural-urban linkages

Telemedicine for specialist support

Rural connectivity focus

Government + NGOs

Improved rural access & diagnostics

https://health.go.ug/programs/digital-health

Ukraine

Lower-Middle

eHealth system (e.g., eHealth platform for primary care, Diia app integration)

Government-led with private involvement

Standards-based, patient-centered records

Rapid digital transformation post-2017

Government + donors (e.g., World Bank, USAID)

Advanced in primary care; expanded during conflict for service continuity

https://ehealth.gov.ua

Uzbekistan

Lower-Middle

National Digital Health Platform (DHP) under "Digital Uzbekistan-2030"

Government-led, standards-based

SNOMED CT adoption, interoperability layer

Nationwide ecosystem build

Government + SNOMED International, donors

Accelerating; unified platform for registries, secure exchange

https://digital.uz
